# Antiphospholipid antibodies increase the levels of mitochondrial DNA in placental extracellular vesicles: Alarmin-g for preeclampsia

**DOI:** 10.1038/s41598-017-16448-5

**Published:** 2017-11-29

**Authors:** Mancy Tong, Caroline Johansson, Fengyi Xiao, Peter R. Stone, Joanna L. James, Qi Chen, Lynsey M. Cree, Lawrence W. Chamley

**Affiliations:** 1 Department of Obstetrics and Gynaecology, School of Medicine, The University of Auckland, Auckland, 1023 New Zealand; 2 Faculty of Medicine and Health Sciences, Linköping University, Linköping, SE-581 83 Sweden; 3 The Hospital of Obstetrics & Gynaecology, Fudan University, Shanghai, China

## Abstract

The pathogenesis of preeclampsia remains unclear but placental factors are known to play a crucial role causing maternal endothelial cell dysfunction. One potential factor is placental micro- and nano- vesicles. Antiphospholipid antibodies (aPL) increase the risk of preeclampsia ten-fold, in part by damaging the mitochondria in the syncytiotrophoblast. Since mitochondrial DNA (mtDNA) is a danger- associated molecular pattern (DAMP/alarmin) that may activate endothelial cells, the aims of the current study were to investigate whether aPL affect the number of placental vesicles extruded, their mtDNA content and their ability to activate endothelial cells. Exposure of first trimester human placental explants to aPL affected neither the number nor size of extruded micro- and nano- vesicles (n = 5), however their levels of mtDNA were increased (n = 6). These vesicles significantly activated endothelial cells (n = 5), which was prevented by blocking toll-like receptor 9 (TLR-9), a receptor for extracellular DNA. Thus, aPL may increase the risk of preeclampsia in part by increasing the amount of mtDNA associated with placental vesicles. That mitochondrial DNA is recognised as a DAMP by TLR-9 to cause endothelial cell activation, raises the possibility that placental vesicles or TLR-9 might be a target for pharmaceutical intervention to reduce the consequences of aPL in pregnancy.

## Introduction

Preeclampsia is a life-threatening hypertensive disease specific to human pregnancy that affects 3–7% of otherwise healthy pregnant women. While the pathogenesis of preeclampsia is poorly understood, it is clear that factors released by the placenta trigger maternal endothelial cell activation and inflammation early in gestation, leading to the symptoms of this disease^1,2^. The human placenta is covered by a single multinucleated cell, the syncytiotrophoblast, which extrudes a large array of extracellular vesicles (EVs- lipid-enclosed subcellular particles) into the maternal blood. Placental EVs include macro-vesicles (a mixture of multinucleated syncytial nuclear aggregates and other large cellular debris), as well as smaller micro- and nano-vesicles, including exosomes^3,4^. These EVs, especially the smaller micro- and nano- vesicles, are present in the maternal circulation from as early as six weeks of gestation^5,6^ and *in vitro* experiments have reported that they can interact with endothelial cells, monocytes, lymphocytes, neutrophils and platelets^7–13^. In preeclampsia, the number of circulating placental EVs is substantially increased and it has also been suggested that the nature of these EVs may also be altered such that they become dangerous to maternal cells, contributing to the clinical symptoms of this disease^14–18^

Antiphospholipid antibodies (aPL) are autoantibodies that bind to complex antigens including phospholipids and phospholipid-binding proteins, such as β_2_-glycoprotein I^19^. These autoantibodies cause thrombosis and recurrent pregnancy loss; and are the strongest maternal risk factor for preeclampsia, increasing the risk almost ten-fold^20–22^. During pregnancy, aPL have a marked tropism for the placenta and previous work has shown that aPL are rapidly internalised by the syncytiotrophoblast where they caused mitochondrial swelling, inner mitochondrial membrane leak and cytochrome C release into the cytoplasm^23,24^. In most mononuclear cells, mitochondrial dysfunction and release of cytochrome C into the cytoplasm would result in cell death. However, in the multinucleated syncytiotrophoblast, we have previously demonstrated that this leads to the increased extrusion of dangerous macro-vesicles that can subsequently activate endothelial cells^23–25^. Exactly why the extruded macro-vesicles were dangerous and whether aPL also caused changes in the smaller micro- and nano-vesicles are not known.

In addition to being the major powerhouses of a cell, mitochondria contain their own circular DNA (mtDNA) that resembles bacterial DNA^26,27^. When released from the mitochondria, mtDNA can act as a danger-associated molecular pattern (DAMP) and activate intracellular danger-sensing toll-like receptors (TLRs), specifically TLR-9, to induce sterile inflammation^28–33^. Thus, using a well-established placental explant culture model and a well-characterized mouse monoclonal IgG antibody against human β2-glycoprotein I, this study aimed to investigate; 1) whether aPL can affect the amount or size of micro- and nano- vesicles released by first trimester human placentae, 2) whether mtDNA is associated with micro- and nano-vesicles, and 3) whether this mtDNA can contribute to the endothelial cell activation that is characteristic of preeclampsia.

## Results

### Antiphospholipid antibodies did not affect the number of micro- or nano- vesicles released by human placental explants, but human aPL increased the size of nano-vesicles extruded

In order to investigate whether aPL affected the production of micro- or nano- vesicles from human placentae, first trimester placentae were used as this is when the pathology of preeclampsia begins. Placental explants were exposed to either the murine aPL (ID2) or human aPL, and isotype-matched control IgG; and the size and number of micro- and nano- vesicles extruded were quantified by nanoparticle tracking analysis.

**Figure 1:**
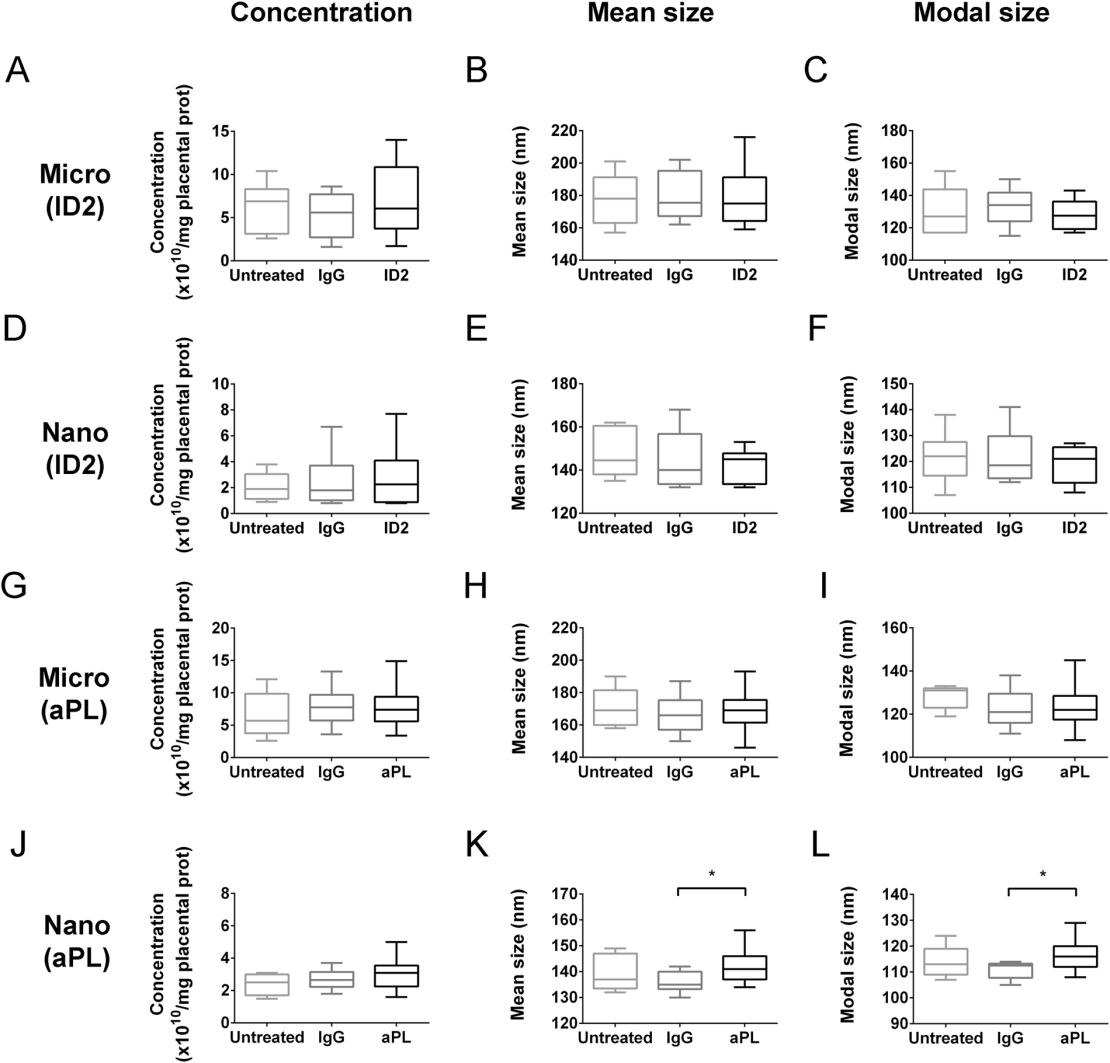
Production of micro- and nano- vesicles by first trimester human placentae in the presence of aPL. First trimester human placentae were cultured with placental culture medium (Untreated), or in the presence of isotype-matched control antibody (IgG) or murine aPL antibodies (ID2, 50 µg/mL, n = 10 placentae, **A**–**F**). In some experiments, human placentae were cultured in the presence of total IgG from aPL-positive patients (aPL, 50 µg/mL, n = 5 placentae), with total IgG from non-autoimmune individuals being used as a control (IgG, **G**–**L**, n = 5 placentae). Micro- (**A**–**C**, **G**–**I**) and nano- (**D**–**F**, **J**–**L**) vesicles were collected, and their concentration, mean and modal size was analysed by nanoparticle tracking analysis. Statistical differences between treatment groups were examined by repeated measures one-way ANOVA with Bonferroni’s correction (*p < 0.05).

ID2 did not significantly alter the amount, mean size or modal size of either micro- or nano- vesicles released from first trimester human placental explants (n = 10, Fig. 1A–F). Human serum-derived aPL also did not significantly affect the number or size of micro-vesicles (n = 5, Fig. 1G–I) nor the number of nano-vesicles released from first trimester human placental explants (n = 5, Fig. 1J). However, exposure to human aPL significantly increased the mean and modal sizes of the extruded nano-vesicles, compared to that from placental explants cultured with control human IgG (p < 0.05, n = 5, Fig. 1K–L).

### Extracellular vesicles extruded from aPL-exposed placental explants activated endothelial cells

**Figure 2:**
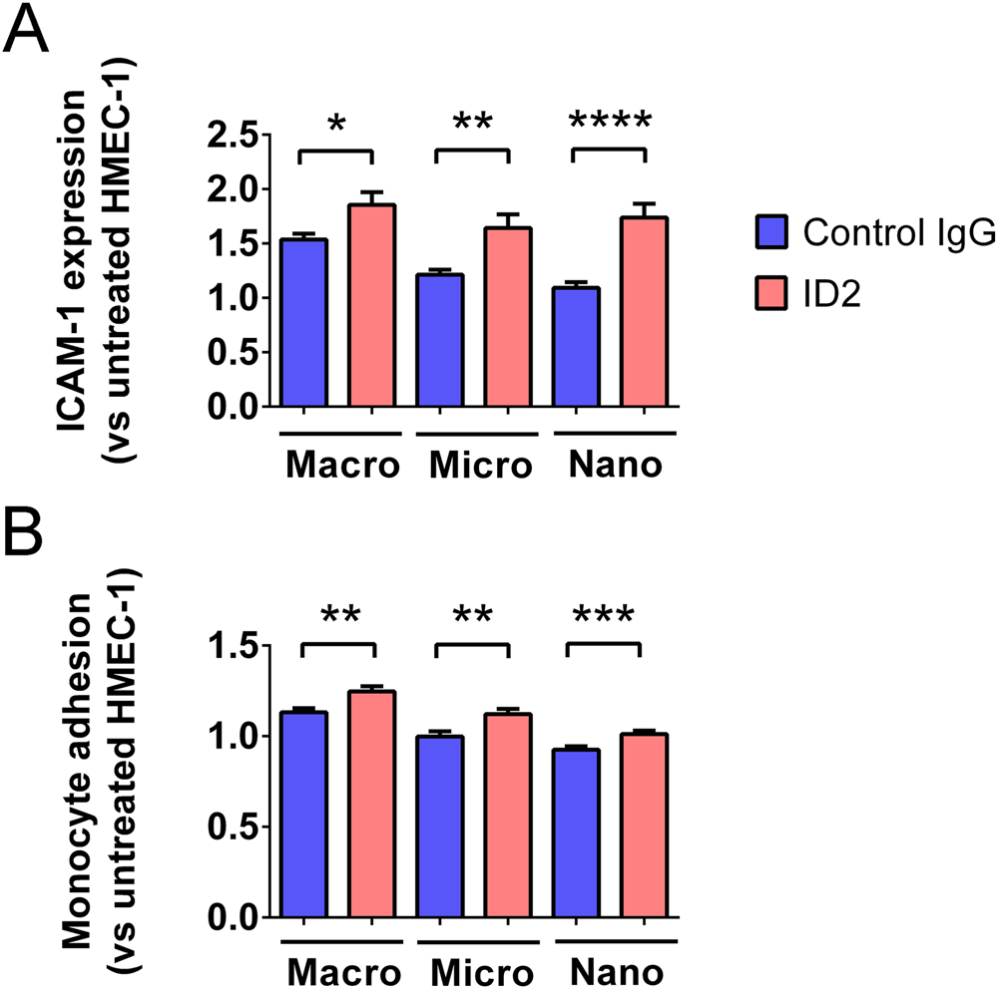
Levels of surface ICAM-1 expression and monocyte adhesion by HMEC-1 cells after co-culture with EVs from ID2-treated placentae. Macro-, micro- and nano- vesicles were collected from first trimester human placentae that have been cultured with ID2 antibodies or isotype-matched control IgG (50 µg/mL, n = 5 placentae). Each fraction of EVs was added to confluent HMEC-1 cell monolayers for 24 hours and endothelial cell activation was quantitated by cell-based ELISA of ICAM-1 expression (**A**) or using the monocyte adhesion assay (**B**). Statistical differences were assessed by Mann-Whitney tests (*p < 0.05, **p < 0.01, ***p < 0.001, ****p < 0.0001, mean ± SEM).

Since we have previously shown that aPL induced the release of dangerous macro-vesicles from the placenta, the current study investigated whether aPL can also induce the release of dangerous placental micro- or nano- vesicles that have the capacity to activate endothelial cells. All three sizes of EVs extruded from ID2-exposed placental explants significantly increased endothelial surface ICAM-1 expression (p < 0.05, n = 5, Fig. 2A) and monocyte adhesion (p < 0.01, n = 5, Fig. 2B) compared to EVs from control IgG-treated placental explants.

### Micro- and nano- vesicles did not carry markers of intact mitochondria or nuclei

Placental macro-vesicles are large and contain many intact nuclei and mitochondria. However, it remains unclear if the much smaller micro- and nano- vesicles might also contain whole mitochondria or nuclei. Therefore, western blots were used to examine whether complex IV, an established mitochondrial marker, and lamin B, an established nuclear marker were present in these smaller EVs.

**Figure 3:**
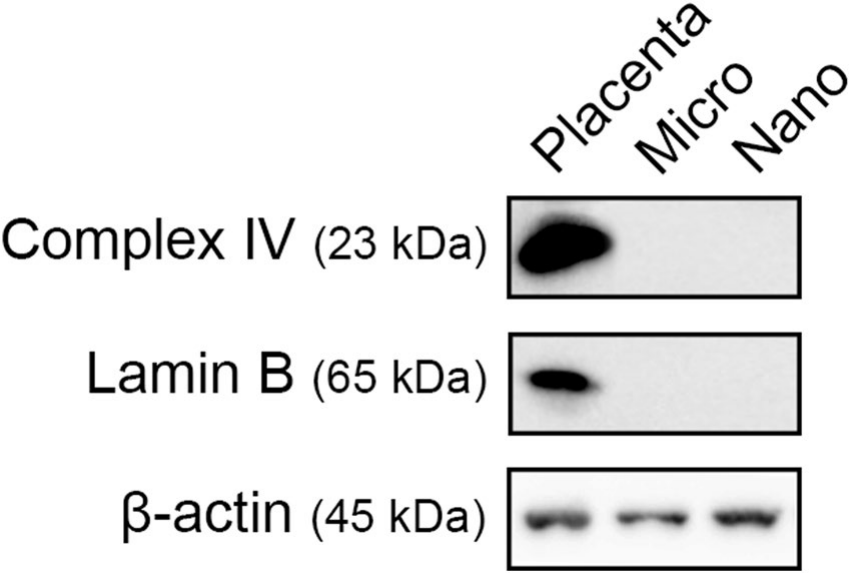
Representative western blots of mitochondrial and nuclear proteins in placental micro- and nano- vesicles. Total protein from micro- and nano- vesicles from first trimester human placentae was extracted and the presence of complex IV, lamin B and β-actin was interrogated by western blotting (n = 6 placentae). Placental lysates were included as a positive control.

Positive control lysates of placental explants contained both mitochondrial complex IV and nuclear lamin B, while placental micro- and nano- vesicles were not positive for either marker (n = 6, Fig. 3). This suggests the absence of intact nuclei and mitochondria in the placental micro- and nano- vesicle fractions.

### Placental EVs carry both nuclear and mitochondrial DNA

**Figure 4:**
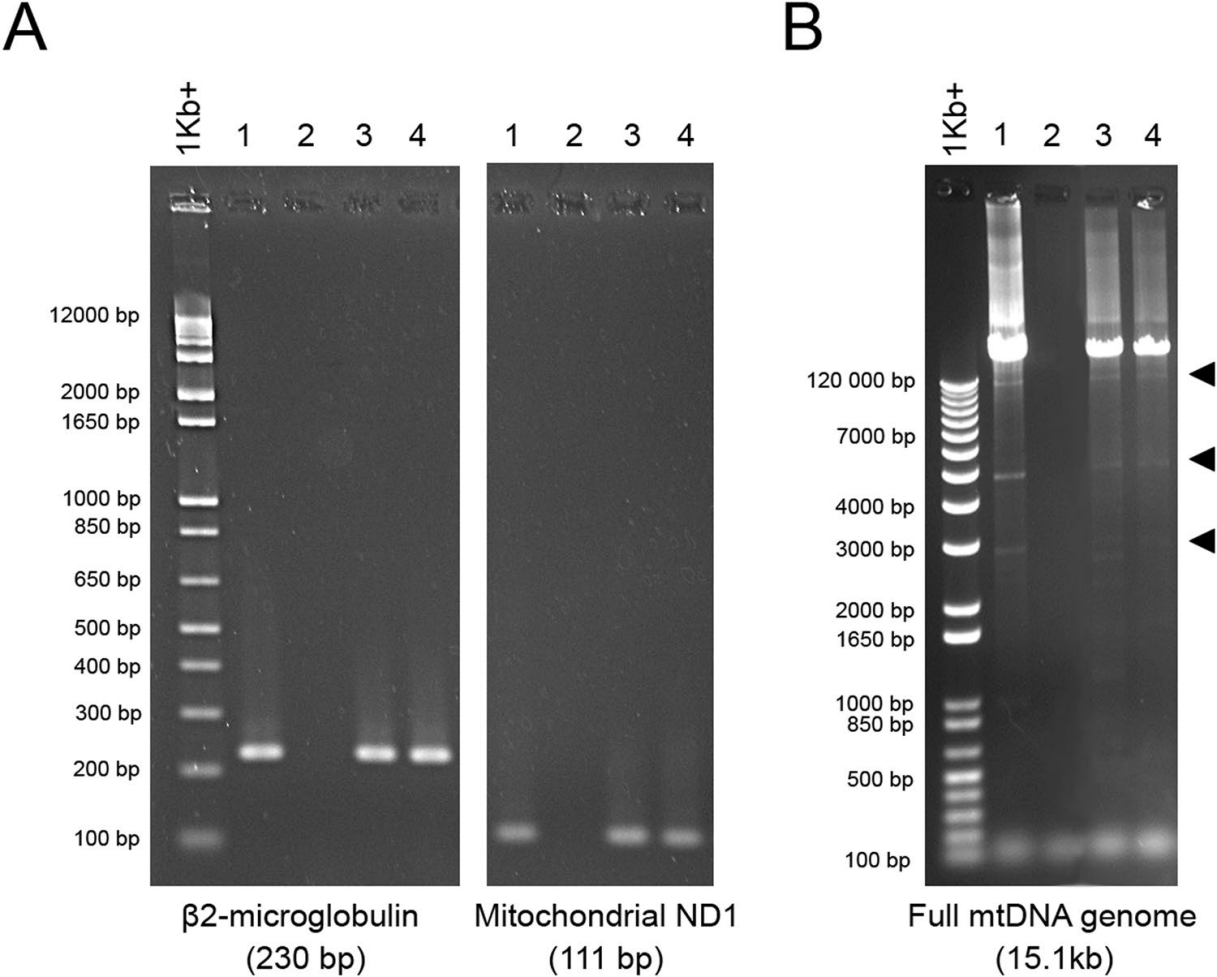
Representative gel electrophoresis images of nuclear and mitochondrial DNA PCR amplicons. Total DNA was extracted from micro- and nano- vesicles from first trimester human placentae (n = 5 placentae). Standard PCR for nuclear β2-microglobulin and mitochondrial ND1 was performed and the products visualised on 2.5% agarose gels (**A**). Long-range PCR for the full conserved sequence of the mitochondrial genome was performed and the resultant amplicons were visualised on 0.7% agarose gels (**B**). The main amplicon detected was 15.1 kb, in addition to several smaller amplicons of mtDNA fragments (arrowheads). 1: First trimester placenta, 2: No template control, 3: Micro-vesicles, 4: Nano-vesicles.

The mean levels of DNA in macro-, micro- and nano- vesicles extruded from first trimester human placental explants were 186.8 ± 63.7 ng, 396.6 ± 92.5 ng and 556.7 ± 81.5 ng per gram of originating placental tissue, respectively (mean ± SEM, n = 7 placentae). In order to determine whether this DNA is mitochondrial or nuclear in nature, PCR for ND1, a mitochondrial-encoded gene, and β2- microglobulin, a nuclear gene, was performed. Both ND1 and β2- microglobulin were detected in micro- and nano- vesicles from first trimester human placental explants (n = 5, Fig. 4A).

Mitochondrial DNA (mtDNA) is circular and approximately 16.5 kb long. Long-range PCR confirmed that both the full-length (excluding the hypervariable region), and smaller fragments of the mitochondrial genome (12.9 kb, 5.1 kb, 2.8 kb) were present in placental micro- and nano- vesicles (n = 5, Fig. 4B).

### Micro- and nano- vesicles from aPL-exposed placental explants had higher levels of mitochondrial, but not nuclear, DNA

In order to investigate whether aPL can affect the amount of mitochondrial and nuclear DNA packaged into placental micro- and nano- vesicles, first trimester human placental explants were cultured with ID2 or control IgG (50 µg/mL) for 18 hours. The absolute copy numbers of mitochondrial and nuclear DNA present in the extruded micro- and nano- vesicles were quantified by quantitative digital PCR for ND1 and RNase P, respectively.

**Figure 5:**
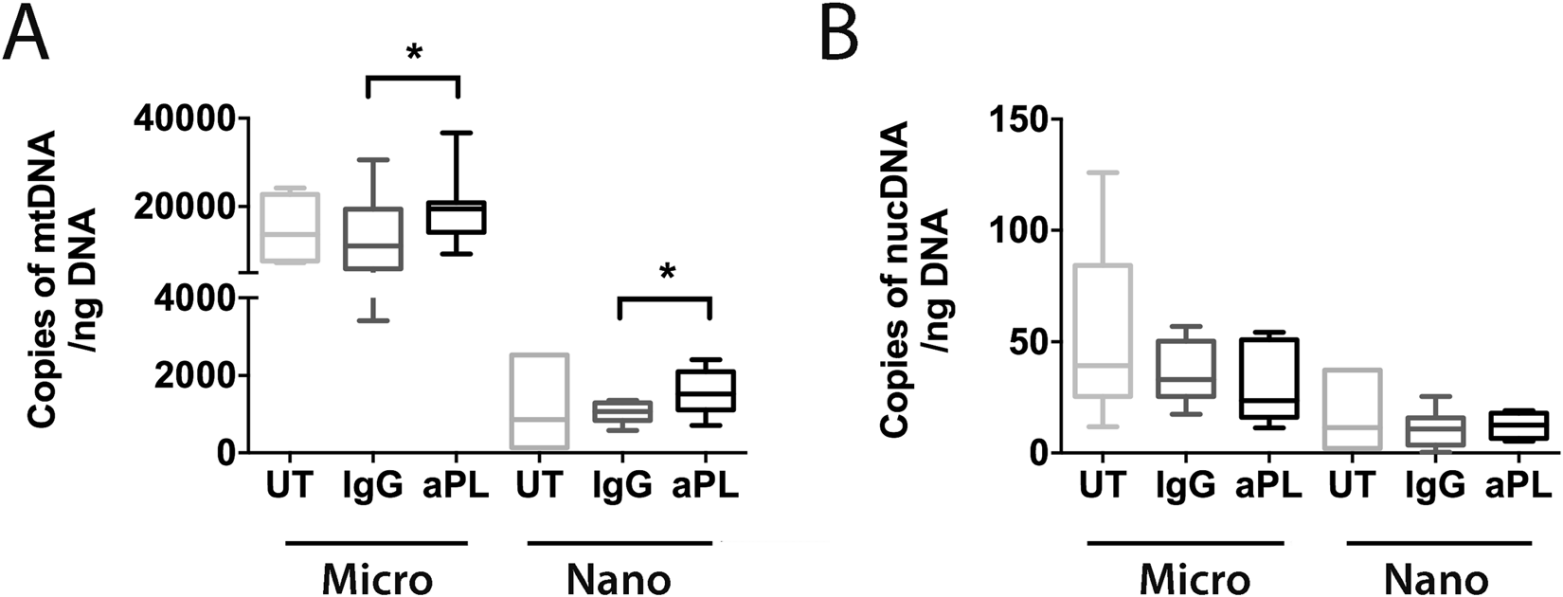
Levels of mitochondrial and nuclear DNA in micro- and nano- vesicles from ID2-treated placentae. Total DNA was extracted from micro- and nano- vesicles from first trimester human placentae that were cultured in the absence of antibodies (UT), or in the presence of isotype-matched control IgG (IgG) or ID2 antibodies (50 µg/mL, n = 6 placentae). Quantitative digital PCR using probes against ND1 and RNase P was performed. The absolute copy number of mtDNA (**A**) and nuclear DNA (**B**) in one nanogram of total DNA is depicted. As data was normally distributed, a repeated measures one-way ANOVA was performed between treatment groups to test for statistical differences (*p < 0.05, **p < 0.01).

Treatment of placental explants with ID2 significantly increased the copy numbers of mtDNA present in both micro- and nano- vesicles extruded from one gram of placental tissue compared to treatment with control IgG (micro-vesicles: 19,426 ± 2859 vs. 13,345 ± 3563 copies; nano-vesicles: 1568 ± 243 vs. 1046 ± 113 copies, n = 6, Fig. 5A). Conversely, the copy numbers of nuclear DNA carried by micro- and nano- vesicles were not significantly altered by treatment of placental explants with ID2 (n = 6, Fig. 5B).

### Micro- and nano- vesicles can interact with lysosomes and activate TLR-9 in endothelial cells

**Figure 6:**
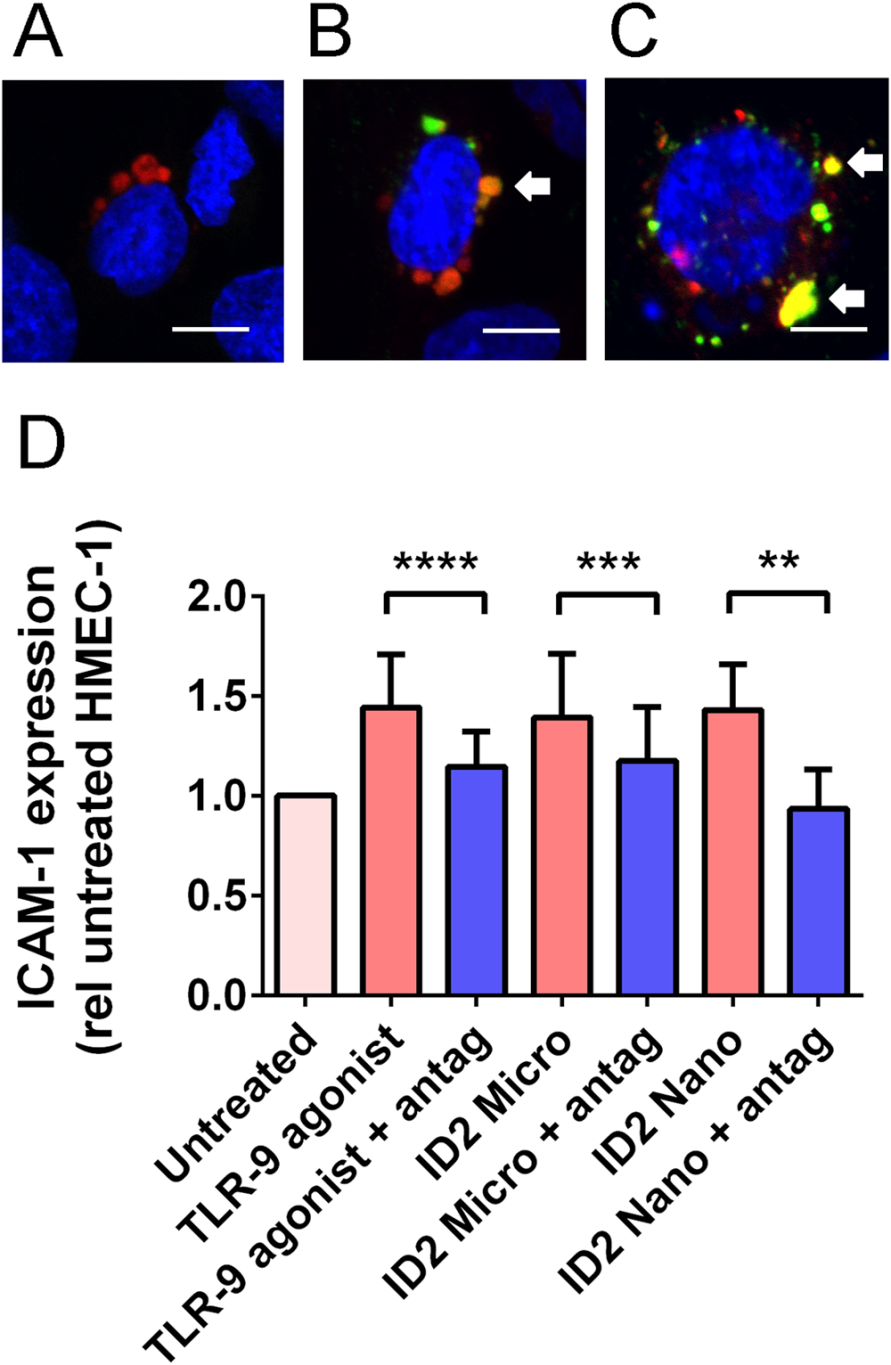
Interactions between placental EVs and TLR-9 of HMEC-1 cells. Optical sections by fluorescence confocal microscopy showing HMEC-1 cells (**A**) that have been cultured with CellTracker^TM^ Green CMFDA-labelled placental micro- (**B**) or nano- (**C**) vesicles (green). Lysosomes of HMEC-1 cells were labelled with LysoTracker® Red DND-99 (red) and the nuclei of HMEC-1 cells were counterstained with Hoechst (blue). Arrows show areas of co-localisation between placental EVs and lysosomes. Scale bar = 10 µm. Micro- and nano- vesicles collected from ID2-treated placentae were added to HMEC-1 cells with or without a TLR-9 antagonist (1 µM, antag, n = 6 placentae). As a positive control, HMEC-1 cells were treated with a TLR-9 agonist (5 µM). Surface ICAM-1 expression was measured by cell-based ELISA and normalised to that of untreated HMEC-1 cells (**D**). Statistical difference was assessed by Wilcoxin matched-pairs signed ranked tests (**p < 0.01, ***p < 0.001, ****p < 0.0001).

Since we have recently shown that placental micro- and nano- vesicles can be internalised by endothelial cells^34,35^, the mtDNA associated with placental EVs could potentially act as a danger-associated molecular pattern (DAMP) and activate TLR-9 in endothelial cell lysosomes. In order to investigate whether placental EVs can interact with endothelial cell lysosomes, placental EVs were labelled with CellTracker^TM^ Green and co-cultured with HMEC-1 endothelial cells that were labelled with LysoTracker® Red and Hoechst nuclear stain. Confocal microscopy showed partial co-localisation of placental EVs with endothelial cell lysosomes (n = 3, Fig. 6A–C).

In order to investigate whether micro- and nano- vesicles from aPL-exposed placental explants induced endothelial cell activation via TLR-9, HMEC-1 cells were cultured with micro- and nano- vesicles extruded from ID2-exposed placental explants, in the presence or absence of a TLR-9 antagonist. Exposure of HMEC-1 cells to micro- or nano- vesicles from ID2-exposed placental explants significantly increased endothelial ICAM-1 expression, to a similar level as that induced by a TLR-9 agonist (p < 0.01, n = 6, Fig. 6D). This EV-induced increase in endothelial ICAM-1 expression was blocked by co-incubation with a TLR-9 antagonist (p < 0.0001, n = 8, Fig. 6D).

## Discussion

Antiphospholipid antibodies are a major risk factor for the development of obstetric complications, such as recurrent miscarriage and preeclampsia^22,36–38^. Many studies have reported that aPL can directly affect trophoblast function, as systematically reviewed in^39^, and we have previously published that aPL increase the extrusion of dangerous macro-vesicles from the placenta^23–25^. However, whether aPL can also affect the extrusion of micro- and nano- vesicles from the placenta has not yet been determined. Using a minimally-disruptive placental explant culture method, this study showed that while aPL did not affect the number of micro- or nano- vesicles extruded from the placenta, the downstream effects of the extruded EVs were altered such that they induced endothelial cell activation, in part through TLR-9. This may be due to the increased levels of mtDNA present in micro- and nano- vesicles from aPL-exposed placentae which can activate TLR-9. Thus, this work supports the hypothesis that mtDNA may be one significant danger signal/alarmin carried by EVs from aPL-exposed placentae that can cause endothelial cell dysfunction, potentially contributing to the increased risk of preeclampsia in women with aPL.

Our use of a minimally-disruptive method for generating the EVs in this study is important as it has been reported by others that the method used to prepare placental EVs can significantly impact on the downstream effects of the EVs on target cells^40,41^. In the current literature, the placental explant culture and perfusion methods are accepted to produce the most physiologically relevant EVs, mimicking the EVs extruded by the placenta *in vivo* ^40–43^.

The formation of placental EVs, especially macro- and micro- vesicles, may be part of the programmed cell death process^44–46^. Previous work has shown that exposure of placentae to aPL increased the extrusion of macro-vesicles by over 50%^23,25^. Therefore, it was surprising to observe that in the same experimental system, the number of micro- and nano- vesicles extruded from aPL-exposed placentae was not significantly altered. The lack of increase in micro- and nano- vesicles production in the presence of increased macro-vesicle production supports the concept that the mechanisms governing the formation of various types of EVs are different^47,48^. Nevertheless, it was interesting to observe that the average size of nano-vesicles extruded from placentae exposed to patient aPL was increased compared to those from placentae exposed to control IgG (Fig. 1K–L). Larger EVs have been suggested to be more proinflammatory than smaller EVs, therefore, the larger EVs observed to be extruded by aPL-exposed placentae may have different downstream effects compared to those from normal placentae. However, as we used aPL-containing IgG fractions from patients rather than purified aPL, despite using IgG fractions lacking aPL as controls, we cannot rule out the possibility that these effects were induced by other antibodies contained in the IgG fractions. Thus, downstream studies were conducted using the well-characterized monoclonal aPL, ID2.

All fractions of EVs extruded from aPL-exposed placentae can activate endothelial cells. This corroborates and extends upon a previous finding which demonstrated that macro-vesicles extruded from aPL-exposed placentae activated endothelial cells^25^. At this time, we do not know whether aPL associate with the extruded placental micro- or nano-vesicles, however in our previous studies, we have shown that the dose of aPL used in this work was not sufficient to induce activation of resting endothelial cells^49,50^. Endothelial cell activation is a central hallmark of preeclampsia that can be detected prior to the clinical symptoms of this condition^51–53^ and this may be one of the mechanisms by which aPL increase the risk of preeclampsia. While aPL did not affect the amount of placental micro- or nano-vesicles produced, these antibodies did alter the nature of the extruded placental EVs such that the EVs conveyed signals of a damaged/stressed placenta to the mother. This feto-maternal signalling is likely to be mediated by changes to the cargo of placental EVs. Supporting this hypothesis, it has been reported that the proteome of macro-vesicles extruded from aPL-exposed placentae is altered compared to that of macro-vesicles from IgG-exposed placentae^23^.

As aPL can be rapidly internalised by the syncytiotrophoblast and induce mitochondrial swelling and dysfunction^23,24^, we were curious to investigate whether aPL increased the packaging of mtDNA into EVs. For this work, only micro- and nano-vesicles were investigated as macro-vesicles are known to contain whole mitochondria, while the smaller EVs did not contain the mitochondrial marker, complex IV, suggesting that these smaller EVs did not contain intact mitochondria nor were they contaminated by mitochondria. For the first time, the data has demonstrated that both intact and fragmented mtDNA is packaged into placental micro- and nano-vesicles, and that the amount of mtDNA present in placental EVs is increased when the placenta has been exposed to aPL. The mechanism(s) by which mtDNA levels are increased in micro- and nano- vesicles from aPL-exposed placentae remains unknown. However, since these antibodies cause mitochondrial swelling and membrane leak in the syncytiotrophoblast, it is possible that these changes may result in mitochondrial rupture and release of mtDNA, allowing this mtDNA to be subsequently packaged into EVs^24^. That the copy number of nuclear DNA did not increase in micro- or nano- vesicles after exposure of the placenta to aPL confirms that there is a specific effect of these antibodies on the syncytiotrophoblast mitochondria.

In addition to being pro-inflammatory, mtDNA can also increase endothelial permeability^29^ and has been suggested to contribute to the development of preeclampsia^54,55^ and intrauterine growth restriction^56,57^. This hypothesis is supported by the observation that the *in vivo* administration of synthetic CpG oligonucleotides, which resemble mtDNA, induced hypertension in pregnant rats^54^, as well as placental necrosis and fetal resorptions in pregnant mice^58^. In addition, this study has now shown that mtDNA packaged in placental micro- and nano-vesicles may also activate endothelial cells through a TLR-9 mediated pathway, supporting the idea that EV-associated mtDNA may be pathological in pregnant women with aPL.

Nuclear DNA is also present in all fractions of placental EVs, albeit at much lower levels than mtDNA. This confirms previous studies which have demonstrated the presence of nuclear DNA in placental micro-vesicles^43,59,60^. It is interesting to note that trophoblast-derived nuclear DNA is less methylated than somatic nuclear DNA, and as such, trophoblast-derived nuclear DNA also has the potential to activate TLR-9^61–63^. Furthermore, trophoblast-derived DNA in preeclampsia may be even more hypomethylated than that from healthy pregnancies^64,65^. Therefore, the presence of nuclear DNA in placental EVs, which are derived from trophoblasts, may also contribute to TLR-9 activation and endothelial cell dysfunction observed in preeclampsia. Whether aPL exposure alters the methylation level of the nuclear DNA packaged in placental EVs requires further investigation.

In light of the current findings, TLR-9 may be a prospective therapeutic target in preeclamptic pregnancies by reducing inflammation and endothelial cell dysfunction. For TLR-9 to be activated, maturation of the endosome containing the free DNA, endosome-lysosome fusion, and binding between the free DNA and TLR-9 must occur. In this work, the addition of a TLR-9 antagonist reduced the endothelial cell activation induced by micro- and nano-vesicles from aPL-exposed placentae *in vitro*, while in pregnant mice it has also been shown that chloroquine can prevent endosomal maturation and reduce DNA-mediated inflammation and pregnancy loss^63^. The prevention of TLR-9 signalling may be one mechanism by which anti-malarial drugs, such as chloroquine and hydroxychloroquine, could possibly exert their beneficial effects in women with preeclampsia or aPL^66,67^.

While it is clear from this work that mtDNA is increased in micro- and nano- vesicles after exposing placental explants to aPL, and that this mtDNA is likely to be major danger signal in these vesicles, this does not rule out the possibility, or likelihood, that these EVs also contain other danger signals. Indeed, we have recently shown that macro-vesicles from aPL-treated placental explants contained elevated levels of HMGB-1, another danger signal^68^. Further work will be required to determine the full burden of danger signals carried by micro- and nano- vesicles from aPL-exposed placentae.

In summary, the current study reported that aPL altered the nature, but not the number, of micro- and nano-vesicles extruded from early gestation human placentae such that they can activate endothelial cells. This work has shown that both mtDNA and nuclear DNA are present in micro- and nano- vesicles from first trimester human placentae and that the packaging of mtDNA is selectively affected by aPL exposure. It seems likely that a major DAMP carried by these EVs is mtDNA which can activate endothelial TLR-9. In addition, trophoblast-derived (hypomethylated) nuclear DNA in placental EVs may also act as a TLR-9-activating DAMP. The combined fetal mitochondrial and nuclear DNA that is associated with EVs from aPL-exposed placentae may represent a strong “alarmin-g” signal that leads to endothelial cell activation, a key step in the pathogenesis of preeclampsia.

## Materials and Methods

### Human ethics approval

Human serum was collected with informed written consent under the approval of the Hospital for Special Surgery Institutional Review Board (Cornell, USA) and the Northern X Regional Ethics Committee (Auckland, NZ). Collection and use of human placentae from Epsom Day Unit, Greenlane Hospital (Auckland, NZ) and Auckland City Hospital (Auckland, NZ) after informed written consent was approved by the Auckland Regional Health and Disabilities Ethics Committee. All experiments were performed in accordance with relevant guidelines and regulations.

### Antiphospholipid and control antibodies

The murine monoclonal anti-β2 glycoprotein I aPL ID2 was generated by hybridoma culture^69^ and purified on HiTrap Protein G columns (GE Healthcare). ID2 is a triple positive antibody that behaves as an anticardiolipin antibody, an anti-β_2_GPI antibody and has lupus anticoagulant activity^70,71^. In all experiments, isotype-matched murine myeloma IgG_1_ antibodies (Invitrogen) were used as a control.

Protein G chromatography was used to purify total IgG fractions from the sera of five patients who were diagnosed with antiphospholipid syndrome and two non-autoimmune individuals, as previously described^24^. All five patients had anticardiolipin IgG > 40GPL and tested positive for anti-β_2_GPI antibodies^24^.

### Collection of placental EVs

Placental EVs were collected from cultured human placentae as previously described^4,12^. Briefly, 400 mg explants were dissected from the villous placenta and cultured in Netwell^TM^ inserts (Corning) in Advanced DMEM/F12 medium supplemented with 2% FBS and 1% Penicillin/Streptomycin (Invitrogen). In some experiments, ID2 (50 μg/mL), isotype-matched control antibody (50 μg/mL), patient aPL (50 μg/mL), control IgG (50 μg/mL), or a fluorescent dye CellTracker® Green CMFDA (2 µg/mL, Invitrogen) was added into the culture medium. After 18 hours of culture at 37 ^o^C with 5% CO_2_/95% air, the culture medium was aspirated and placental EVs of different sizes was separated by sequential centrifugation at 4 ^o^C at 2,000 g for five minutes (macro-vesicles), 20,000 g for one hour (micro-vesicles) and 100,000 g for one hour (nano-vesicles) (Avanti J30I Ultracentrifuge, JA 30.50 Ti fixed angle rotor, Beckman Coulter). Contaminating red blood cells were removed from the macro-vesicle fraction by hypotonic lysis in ultrapure water (EMD Millipore) and contaminating leukocytes were depleted using anti-CD45 magnetic beads (Dako) per the manufacturer’s instructions. The physical characteristics of these different sized vesicles has recently been extensively characterised^4^.

### Nanoparticle tracking analysis

Micro- and nano- vesicles from one gram of placental tissue were resuspended in 0.2µm-filtered PBS (Sigma-Aldrich) to reach measurable concentrations of 2–15 × 10^8^ vesicles/mL and analysed on an NS300 Nanosight instrument equipped with a 405 nm laser and an sCMOS camera (Nanosight). All automatic settings were applied with viscosity setting at 0.95 cP and temperature at 25 ^o^C. A single measurement consists of three 30-second videos and each sample was measured three times in succession, at camera level 10. The detection threshold was set at 10 and data acquisition and processing was performed using the NTA3.0 software (Nanosight). Only recordings with over 1000 valid tracks/vesicles were included in the analysis.

### Cell culture

The human microvascular endothelial cell line (HMEC-1 cells) was purchased from ATCC (CRL3243) and cultured in MCDB-131 medium supplemented with 10% FBS, 1% L-Glutamine and 1% Penicillin/Streptomycin (Invitrogen). Human U937 monocytes were purchased from ATCC (CRL1593.2) and cultured in Advanced DMEM/F12 medium supplemented with 2% FBS and 1% Penicillin/Streptomycin (Invitrogen). Cells were cultured at 37 ^o^C with 5% CO_2_/95% air.

### Endothelial cell activation

#### Cell-surface ELISA for detection of ICAM-1

Cell surface expression of intercellular adhesion molecule 1 (ICAM-1) is a well-described marker of endothelial cell activation^13,72^. Six thousand HMEC-1 cells were grown to confluence before exposure to placental EVs, with and without a TLR-9 antagonist (5 µM, Invivogen, Table 1), for 24 hours in quadruplicate. Then, cells were washed and a mouse anti-human ICAM-1 antibody (MCA1135, Bio-rad, 1:100) was added and incubated at 37 °C for two hours, followed by a goat biotinylated anti-mouse IgG antibody (Jackson ImmunoResearch, 1:2000) and a streptavidin-conjugated HRP (Jackson ImmunoResearch, 1:2000). o-Phenylenediamine, a substrate for HRP, was added at 20 ^o^C for detection at 490 nm using an xMark spectrophotometer (Bio-rad).

**Table 1:** Details of nucleotides and primers used.

**Name**	**Sequence (5′ to 3′)**
Full 15 kb mtDNA sequence F′	TTA AAA CTC AAA GGA CCT GGC
Full 15 kb mtDNA sequence R′	GTG GGT AGG TTT GTT GGT ATC
NADH dehydrogenase 1 F′	ACG CCA TAA AAC TCT TCA CCA AAG
NADH dehydrogenase 1 R′	GGG TTC ATA GTA GAA GAG CGA TGG
TLR-9 agonist	TCG TCG TTT TCG GCG CGC GCC G (on a phosphorothioate backbone)
TLR-9 antagonist	TTT AGG GTT AGG GTT AGG GTT AGG G (on a phosphorothioate backbone)
β2 microglobulin F′	CAC TGA AAA AGA TGA GTA TGC C
β2 microglobulin R′	AAC ATT CCC TGA CAA TCC C

#### Monocyte adhesion assay

Monocyte adhesion to HMEC-1 cells was assessed using U937 monocytes that had been fluorescently labelled with CellTracker^TM^ Red CMTPX (2 µg/mL). After HMEC-1 cells were exposed to placental EVs in quadruplicate for 24 hours at 37 ^o^C, cells were washed and fluorescently-labelled monocytes were added (5 × 10^3^ monocytes/well of a 96-well plate). Monocytes and HMEC-1 cells were co-cultured for five hours at 37 ^o^C. Then, unbound monocytes were removed and the fluorescence remaining in each well was measured using a Synergy 2 microplate reader (BioTek) at 530/620 nm.

### Protein extraction and western blotting

Total protein from placental EVs were extracted using RIPA buffer (50 mM Tris, 150 mM NaCl, 1% sodium deoxycholate, 0.1% SDS, 1% Nonidet P40 substitute, 1 mM PMSF, pH7.4) supplemented with protease inhibitor (Roche) and resolved by SDS-PAGE. Proteins were transferred onto Hybond^TM^-C extra nitrocellulose membranes (Amersham Biosciences), which were blocked of non-specific binding with 5% non-fat milk powder/PBST. Membranes were then probed with rabbit anti-human complex IV (Thermo Fisher Scientific, A-6404, 1:750) or rabbit anti-human lamin B (Abcam, ab16048, 1:500) antibodies before applying HRP-conjugated anti-rabbit IgG antibodies (Jackson ImmunoResearch, 1:2000). Target proteins were visualised using Amersham^TM^ ECL^TM^ Prime detection reagent on an Image Quant LAS3000 (GE Healthcare) and images were annotated using Adobe® Photoshop® Elements 5.0.

### Detection of DNA in placental EVs

#### Total DNA extraction

Placental EVs were resuspended in Quick-gDNA Miniprep Genomic Lysis Buffer and total DNA was extracted using the Quick-gDNA Miniprep kit (Zymo Research). Extracted DNA was eluted with 50 µL of DNA Elution Buffer for five minutes at 20 ^o^C and stored at −20 ^o^C. Total levels of DNA extracted from macro-, micro- and nano- vesicles were quantified using a Qubit® 3.0 Fluorometer using the Qubit® dsDNA High Sensitivity Assay Kit (Thermo Fisher Scientific).

#### Standard polymerase chain reaction (PCR)

All PCR reactions were prepared in the nucleic acid-free zone of the laboratory. To detect nuclear and mitochondrial DNA, PCR of β2 microglobulin and NADH dehydrogenase 1 (ND1) was performed, respectively. Reactions were prepared on ice containing 1x standard Taq reaction buffer, 200 µM dNTP mix (Roche), 0.2 µM each of forward and reverse primers (Table 1), 0.625U Taq polymerase and ultrapure water to a 25 µL final volume. 50 ng of extracted DNA was added. For all PCR reactions, a negative control where ultrapure water was added instead of a DNA template was included.

Polymerase chain reaction amplifications were performed in an iCycler C1000^TM^ thermal cycler (Bio-rad) following a standard PCR cycling protocol (denaturation at 94 ^o^C for five minutes; 30 cycles of one minute at 94 ^o^C, one minute at 58 ^o^C and one minute at 72 ^o^C; and a final extension cycle at 72 ^o^C for ten minutes).

#### Long-range PCR

The presence of intact mtDNA genomes was investigated by long-range PCR. Reactions were prepared on ice containing 1x reaction buffer, 1.6 mM dNTP mix, 0.2 µM each of forward and reverse primers which amplified the whole conserved sequence of the mtDNA genome (Table 1), 2.5U TaKaRa® Taq polymerase (ClonTech) and ultrapure water to a final volume of 50 µL. Finally, 50 ng of extracted DNA was added. For all PCR reactions, a negative control was included. The PCR cycling protocol used was one minute at 94 ^o^C; 30 cycles of 30 seconds at 94 ^o^C, 30 seconds at 62 ^o^C, 16 minutes at 68 ^o^C; and 15 minutes at 72 ^o^C.

#### Gel electrophoresis

Electrophoresis of amplicons from standard PCRs were performed on 1.5% agarose gels (w/v) while amplicons from long-range PCRs were resolved on 0.7% agarose gels. Gels were stained in ethidium bromide and visualised under UV light using a GelDoc^TM^ XR system (Bio-rad). Molecular weights of the PCR amplicons were estimated from the 1 kb+ molecular weight ladder included in a lane parallel to the samples.

### Digital PCR

The absolute copy number of nuclear and mitochondrial DNA present in placental micro- and nano- vesicles was determined using a QuantStudio 3D Digital PCR System (Thermo Fisher Scientific). After initial multiplex validation, a single copy nuclear gene (ribonuclease P [RNAse P]), and a mitochondrial gene (ND1) were simultaneously targeted for absolute quantification. A TaqMan copy number reference assay for RNAse P was used, which included a VIC dye-labelled TAMRA probe (catalogue ID: 4403326), while a TaqMan copy number assay was customised for ND1, which included a FAM dye-labelled MGB probe (assay ID: MT-ND1_CCCSVXC; Applied Biosystems).

After initial optimisation to prevent saturation, 0.3 ng of total DNA from placental micro-vesicles was loaded, while 3 ng of total DNA from placental nano-vesicles was loaded. The digital PCR reaction mixture consisted of the QuantStudio 3D digital PCR mastermix, TaqMan copy number assay probes and the aforementioned amount of purified DNA, made up to a final volume of 15 μL using ultrapure PCR-grade water. All reactions were prepared in a laminar PCR flow hood to minimise DNA contamination. A no-template control chip was also included for every batch of samples to detect contamination.

Polymerase chain reaction was performed in the ProFlex 2x Flat PCR System with the cycling conditions set at 96 °C for ten minutes, followed by 39 cycles of 60 °C for two minutes and 98 °C for 30 seconds, and a final extension at 60 °C for two minutes. Following PCR, image capture and measurement of end-point fluorescence was performed on a QuantStudio 3D Digital PCR Instrument (Thermo Fisher Scientific). Downstream analysis was performed on the QuantStudio 3D Analysis Suite Cloud Software according to the manufacturer’s instructions^73^. For RNAse P, the fluorescence threshold was set to 4000 while for ND1, 2500 was used. The measured number of DNA copies was normalised to the amount of template DNA loaded (either 0.3ng or 3ng for placental micro- and nano- vesicles, respectively).

### Visualisation of interaction between placental EVs and endothelial cells

Human microvascular endothelial cells were grown on coverslips until confluent. Then, CellTracker^TM^ Green CMFDA-labelled placental EVs were added and co-cultured with HMEC-1 cells for 18 hours. Coverslips were washed and LysoTracker^TM^ DND-99 (75pM, Thermo Fisher) was added for one hour at 37 ^o^C. Nuclei were counterstained with Hoechst (100 µg/mL) prior to fixation with 4% PFA (Sigma-Aldrich) for ten minutes at 20 ^o^C. Cells were visualised on the Olympus FV1000 confocal fluorescence microscope.

### Statistical analysis

All data were paired, as explants from the same placenta were treated with either aPL/ID2 or control IgG antibodies to reduce intrinsic variability between placentae. Data was tested for normality by the Shapiro-Wilk normality test. For parametric data, the paired t-test or the repeated measures one-way ANOVA was used to assess statistical significance on GraphPad PRISM 6.01 (GraphPad Software Inc). For non-parametric data, the Wilcoxon matched-pairs signed rank test or the Friedman test for multiple comparisons was used to assess statistical difference as appropriate. An adjusted p value < 0.05 was considered statistically significant.
